# Registered access: authorizing data access

**DOI:** 10.1038/s41431-018-0219-y

**Published:** 2018-08-02

**Authors:** Stephanie O. M. Dyke, Mikael Linden, Ilkka Lappalainen, Jordi Rambla De Argila, Knox Carey, David Lloyd, J. Dylan Spalding, Moran N. Cabili, Giselle Kerry, Julia Foreman, Tim Cutts, Mahsa Shabani, Laura L. Rodriguez, Maximilian Haeussler, Brian Walsh, Xiaoqian Jiang, Shuang Wang, Daniel Perrett, Tiffany Boughtwood, Andreas Matern, Anthony J. Brookes, Miro Cupak, Marc Fiume, Ravi Pandya, Ilia Tulchinsky, Serena Scollen, Juha Törnroos, Samir Das, Alan C. Evans, Bradley A. Malin, Stephan Beck, Steven E. Brenner, Tommi Nyrönen, Niklas Blomberg, Helen V. Firth, Matthew Hurles, Anthony A. Philippakis, Gunnar Rätsch, Michael Brudno, Kym M. Boycott, Heidi L. Rehm, Michael Baudis, Stephen T. Sherry, Kazuto Kato, Bartha M. Knoppers, Dixie Baker, Paul Flicek

**Affiliations:** 1 Centre of Genomics and Policy, Faculty of Medicine, McGill University, Montreal, QC Canada; 2 CSC – IT Center for Science, Espoo, Finland; 3 ELIXIR Hub, Wellcome Genome Campus, Hinxton, Cambridge, UK; 4 European Molecular Biology Laboratory, European Bioinformatics Institute, Hinxton, UK; 5 Centre for Genomic Regulation, Barcelona, Spain; 6 Universitat Pompeu Fabra, Barcelona, Spain; 7 The Global Alliance for Genomics and Health, MaRS Centre, West Tower, 661 University Avenue, Suite 510, Toronto, M5G 0A3 ON Canada; 8 Broad Institute of MIT and Harvard, Cambridge, MA USA; 9 Wellcome Trust Sanger Institute, Wellcome Genome Campus, Hinxton, Cambridge, UK; 10 Center for Biomedical Ethics and Law, Department of Public Health and Primary Care, University of Leuven, Leuven, Belgium; 11 National Human Genome Research Institute, NIH, Bethesda, MD USA; 12 Genomics Institute, University of California at Santa Cruz, Santa Cruz, CA USA; 13 OHSU, Portland, OR USA; 14 Department of Biomedical Informatics, UC San Diego, La Jolla, CA USA; 15 Australian Genomics Health Alliance, 50 Flemington Road, Parkville, VIC 3052 Australia; 16 Bioreference Laboratories, Inc., Elmwood Park, NJ USA; 17 Department of Genetics and Genome Biology, University of Leicester, Leicester, UK; 18 DNAstack, Toronto, ON Canada; 19 Microsoft, Redmond, WA USA; 20 Google, Mountain View, CA USA; 21 McGill Centre for Integrative Neurosciences, Montreal Neurological Institute, McGill University, Montreal, QC Canada; 22 Vanderbilt University Medical Center, Nashville, TN USA; 23 UCL Cancer Institute, University College London, London, UK; 24 Department of Plant & Microbial Biology, University of California, Berkeley, CA USA; 25 ELIXIR Compute Platform, ELIXIR, Wellcome Genome Campus, Hinxton, Cambridge, UK; 26 Department of Computer Science, Biomedical Informatics, ETH Zurich, Zurich, Switzerland; 27 Department of Computer Science, University of Toronto, Toronto, ON Canada; 28 Centre for Computational Medicine, Hospital for Sick Children, Toronto, ON Canada; 29 Children’s Hospital of Eastern Ontario Research Institute, University of Ottawa, Ottawa, ON Canada; 30 Department of Pathology, Brigham & Women’s Hospital & Harvard Medical School, Boston, MA USA; 31 University of Zurich & Swiss Institute of Bioinformatics, Zurich, Switzerland; 32 National Centre for Biotechnology Information, US National Library of Medicine, Bethesda, MD USA; 33 Department of Biomedical Ethics and Public Policy, Graduate School of Medicine, Osaka University, Osaka, Japan; 34 Martin, Blanck & Associates, Alexandria, VA USA; 35 Montreal Neurological Institute, Faculty of Medicine, McGill University, Montreal, QC Canada

## Abstract

The Global Alliance for Genomics and Health (GA4GH) proposes a data access policy model—“registered access”—to increase and improve access to data requiring an agreement to basic terms and conditions, such as the use of DNA sequence and health data in research. A registered access policy would enable a range of categories of users to gain access, starting with researchers and clinical care professionals. It would also facilitate general use and reuse of data but within the bounds of consent restrictions and other ethical obligations. In piloting registered access with the Scientific Demonstration data sharing projects of GA4GH, we provide additional ethics, policy and technical guidance to facilitate the implementation of this access model in an international setting.

## Introduction

As data sharing policies in genomics strive to keep pace with the state of data-intensive science [1, 2], current policies offer little choice for sharing genomic research data beyond the two established mechanisms of *open access*, when data are freely published on the World Wide Web, and *controlled access* (also called *managed* or *restricted access*), whereby qualified researchers apply for access on a project-by-project basis and their research plans are reviewed, often by a committee [3–5]. Both open and controlled access policy models have historically served the research community’s needs, scientific progress and clinical care. However, plans for greater integration of datasets and informatics platforms [6], along with ever greater sharing of health-related datasets and growing interest by clinicians and patients in also accessing genomic data, call for new streamlined models of data access that take greater advantage of the richer access-control policies current technology is capable of enforcing. Access-control policies, and the technology that enforces them, must enable rapid and efficient access to data that is shared only for specific purposes to a wide range of users while effectively managing ethical and legal risks.

## The registered access policy model

Our proposals arise from discussions with a range of stakeholders engaging in international data sharing initiatives as members of the Global Alliance for Genomics and Health (GA4GH) [7]. GA4GH is an international coalition dedicated to improving human health by maximizing the potential of genomic medicine through effective and responsible data sharing, as founded on the *Framework for Responsible Sharing of Genomic and Health-Related Data* [8]. Our work has led us to conclude that there are specific datasets where existing consent agreements and ethical approval are compatible with a novel data access policy model called *registered access* [9]. This model would capitalize on the well-established role-based access control (RBAC) model for information technology security enforcement [10–14] and is based on the notion that potential users could be granted online access to data according to their roles (e.g., bona fide researcher or clinical care professional) and risk analysis, rather than on the basis of a specifically described project as is normally required in the controlled access models commonly implemented for research purposes. RBAC is widely implemented in government and industry throughout the world [15]. By capitalizing on RBAC-based access-control technologies, registered access could, in theory, provide access to all data shared in this way, following a unified general registration process and without the need for individualized data access committee review.

### Examples of registered access

Registration as a means to limit access to data to approved users—albeit with different approval processes—has already been used in several genomics projects. For example, the Wellcome Trust Case-Control Consortium required registration for access to summary allele frequency datasets once it was demonstrated that these data could potentially lead to the re-identification of study participants [16, 17]. While this risk was considered to be low, limiting access to consortium researchers seemed to be a reasonable mitigation strategy at the time and was judged by the Consortium Data Access Committee to be consistent with the participant consent agreements. More recently, the “Bravo” project requires a simple form of registration via logging in to access data. Recent policy recommendations based on risk assessment for such data aim to discriminate between a lower and higher risk of potential resulting harm in the case of re-identification, for example, limiting access to aggregate data according to whether data were associated with more sensitive health or demographic information (e.g., ethnicity information about small or vulnerable populations) [18, 19].

Another current example of a registration-based data access policy is the DatabasE of genomiC varIation and Phenotype in Humans using Ensembl Resources (DECIPHER [20]). Users who have been approved by the project coordinator (a senior physician working at the center depositing the data) are granted registered access to that project data. DECIPHER projects can be linked to form a consortium, allowing intra-consortium sharing. PhenomeCentral is another example of a registered access policy for the identification of additional cases for ultra-rare disorders [21]. Along with DECIPHER, PhenomeCentral is part of the GA4GH Matchmaker Exchange (MME) initiative. PhenomeCentral users are required to be bona fide researchers or clinicians. This is validated through institutional email addresses, as well as through user-provided and publicly available information such as prior publications, scientific activity at conferences identified through web searches, and mention on institutional websites. Users without a scientific track record (e.g., trainees) can be validated by a more senior colleague. Data entered into PhenomeCentral can then be shared either with chosen researchers or with pre-defined groups (consortia) who have leads responsible for approving membership.

In addition to these intra-consortia, coordinator-approved registration policies, several other current projects are providing, or plan to provide, registration-based access to the research community beyond their projects (see Box 1). These resources either grant an account following a review of an applicant’s credentials (based on submitted or public information) or following a simple registration of their identity. All involve online agreement to data use terms and conditions. CAGI, the Critical Assessment of Genome Interpretation, active since 2010, has several tiers of access according to the sensitivity of datasets [22], which are available to registered users ranging from unaffiliated researchers to trainees entering the field and individuals at companies to well-identified accomplished researchers. Vouching (e.g., of a mentor for a student) can also allow appropriate escalation of access.

Box 1 Examples of current projects enabling registration-based access

**Table:** 

*Resource*	*Access requirements*
Critical Assessment of Genome Interpretation (CAGI) https://genomeinterpretation.org	• Review of users • Digital signing of data use agreement
Simons Foundation Autism Research Initiative (SFARI) https://www.nextcode.com/ssc/	• Review of users • Online agreement to data use conditions
mPower Public Researcher Portal [39] http://sagebase.org/research-projects/mpower-researcher-portal/	• Verification of user identity and training • Online agreement to data use conditions
AACR Project GENIE https://www.synapse.org/#!Synapse:syn7222066/wiki/410922	• Verification of user identity and training • Online agreement to data use conditions
Bravo (http://bravo.sph.umich.edu)	• Login with ID provider (Google ID) linked to work email address • Online agreement to data use conditions

### Implementation in GA4GH

Our model of registered access in the GA4GH context comprises a three-stage “Triple-A registration” process (Authentication, Attestation, and Authorization [9]), which aims to ensure both user identification and agreement to a standard set of general responsibilities while considerably simplifying the data access application process. Through the identification and authentication process, the individual provides “proof” that an asserted identity is their own. The attestation process establishes that the potential data user meets the requirements expected by the consent agreements and ethical approval of datasets in question and includes agreement to comply with the terms of data use required of registered users. Finally, authorization is the overall process by which users are granted access to data and permission to perform specific actions. We provide concrete examples of, and guidance for, each stage in the process based on three GA4GH Demonstration Projects with which we fleshed out standards that would be broadly applicable.

The Beacon Project (manuscript in press), the Matchmaker Exchange [23], and the BRCA Challenge (manuscript submitted) are among the initial demonstration projects that aimed to drive learning, identify requirements, assess value, and coordinate activity within the first phase of GA4GH. For each of these, we explored options for using registered access to improve and streamline access to data that had previously been available either through a controlled access application process and/or bound by protocol-specific restrictions.

For the Beacon Project, which enables the discovery of genetic variants across multiple world-wide datasets, registered access is envisaged as a means to share more details than simple existence of genetic variants (e.g., that they are present in individuals with a specific health condition). In conjunction with Beacon partners ELIXIR (Europe’s infrastructure for life science information) and NCBI (the US National Center for Biotechnology Information), registered access is being developed for access to appropriate metadata from controlled access datasets. Such metadata access is similar to current access protocols at dbGaP [25] and the European Genome-phenome Archive (EGA) [26]. Specifically, users with an eRA account (for the NIH Commons research grant system) are dbGaP registered users with access to some information about available controlled access datasets [24]. Similarly, EGA users who have obtained access to at least one EGA controlled access dataset have access to specific EGA information about available controlled access datasets after logging in with their EGA account.

The MME is a federated network connecting databases of genomic and phenotypic data using a common application programming interface to facilitate rare disease gene discovery, including from DECIPHER (open subset), the PhenomeCentral platform [21], GeneMatcher [27], MyGene2 [28], Patient Archive (*patientarchive.org*), and *matchbox*. In its current iteration, it requires two-sided inquiry (i.e., a search from two parties with a similar patient) and, in this way, connects two investigators looking for a match for the same candidate gene and disease. Each user must be registered in one of the databases in order for data to be deposited and queries made. Future iterations of MME will expand functionality and facilitate a one-sided inquiry, with bona fide investigators identified by a registered access process able to see details of a matched case, including variants in a specific gene and high-level phenotypic information for their purposes as a scientific investigator working to understand the causes of rare diseases.

The goal of the BRCA Challenge is to translate the rapid expansion of sequencing capacity into useful knowledge and, in particular, learn how to rapidly interpret variant data to generate clinical utility. Its intent is to provide an umbrella under which many groups can collaborate and bring together data to improve the precision of assessing variants across both BRCA1 and BRCA2. While its main resource on BRCA variant interpretation is publicly available, overlaying registered access would allow enrichment of the dataset with data that cannot be shared openly: for example, patient data supporting clinical interpretations of variants may not be consented for open release but would be available to expert review teams, researchers, or clinicians.

To support these pilot implementations of registered access in GA4GH, we expand on our initial ethical–legal feasibility study and review of projects that are pioneering registration-based access policy (see Box 1) to describe plans for an international, unified approach that could lead to a standardized registration process allowing for access to a wide range of data resources. All three stages of registered access (authentication, attestation, and authorization) pose significant ethical–legal and technical challenges, which we attempt to address by providing policy and technical guidance.

## Authentication

A potential advantage of the registered access policy model is to efficiently provide data access to a relatively large number of authorised individuals and alleviate the considerable administrative burden on data custodians of managing controlled access requests. This model is premised on the trust that broad categories of registered users, such as researchers and clinical care professionals, will use the data accessed with the same appropriate care as they would manage controlled access data. Defining categories of users as bona fide researchers or clinical care professionals in this context rests largely on the information provided at the time of registration (user attributes) and the attestation they agree to. The attributes requested from users for the registration process, and particularly their verification, will have important implications for access to data protected by registered access authorization methods.

Based on an ethical–legal analysis of research ethics and other legal and administrative frameworks applicable to data sharing and access, it was previously proposed that several elements of controlled access review should be retained in registered access, including for how users might be authorized based on their “competence.” We considered whether it might be necessary to set a few differing levels of stringency for the registered access model (e.g., Registered, Registered+) to cater to different projects’ views of the requisite access and data sensitivity. However, we agreed that a minimal standard (basic registration criteria) could be established, thereby enabling mutual recognition between registration systems established in different parts of the world (e.g., ELIXIR and NCBI). This does not preclude policies that provide different levels of access to data to different categories of users. Indeed, such policies are enforceable using a combination of RBAC and attribute-based access control.

To qualify as either a bona fide researcher or clinical care professional, first of all, individuals will need to provide the following details of their identity and research/clinical activity: name; title; position; affiliation; and institutional email address, phone number, website, and mailing address. As these details may also be provided by an individual’s organization, they are an important means of strengthening accountability and traceability of registered users and can be simply verified by web searches or calls to institutional switchboards.

We considered additional information that could demonstrate a research user’s professional status such as: researcher identity systems (e.g., ORCID or ISNI); PubMed publication IDs; and researcher accounts such as those with funding agencies (e.g., NIH Commons’ eRA), universities (email addresses or user accounts), and the major public archives (e.g., MyNCBI and PubMed Commons). Evidence of academic publication (in the context of a research position) is typically relied upon in the controlled access application process as an indication of researchers’ ability to use data [29]. However, concern was expressed regarding the value of journal publications and some researcher IDs as an indicator of professional activity, especially current activity. There was also concern about the rise in so-called “predatory” academic journals, leading to publications of dubious quality [30].

We eventually decided on a “layered” registration system whereby bona fide researchers or clinical care professionals could either demonstrate their status directly (by providing evidence of professional status, such as license numbers for clinical care professionals) or alternatively have their status “vouched for” by another registered user within their category (for researchers) or their employing institution (for researchers and clinical care professionals) (see Box 2). One use case for such a voucher approach would be for students or trainees who may have neither professional appointment nor publications, where the expectation would be for an advisor to support the registration.

Box 2 The “layered” registration system. Shows the main routes to user authentication for the categories of bona fide researcher and clinical care professional

**Table:** 

*A person may receive bona fide researcher status if:*
1. Their home institution confirms they are researchers, OR
2. A person who satisfies condition (1) corroborates (“vouches for”) their researcher status (as a reference)
*A person may receive clinical care professional status if:*
1. Their home institution confirms they are clinical care professionals, OR
2. They have a physician or other clinical care professional license (ID/permit number)

### Responsibilities of institutions

Accountability of registered users is central to the registered access model. Within data access policy models, various approaches have been proposed to hold users accountable. One is co-signing of a data access agreement by the (home) institution of the users and recognizing this institution as the ultimate responsible entity. Within this perspective, that institution can be legally held accountable if the researcher or clinician commits any wrongdoing. Although registered access does not require signing such an agreement between the home institution and the data custodians, one could argue that, if any wrongdoing happens, the users’ institution will in all likelihood be contacted and asked to enforce administrative disciplinary measures in an analogous way as is currently done in some cases of scientific misconduct, such as plagiarism or publishing falsified data. In turn, in addition to the attestation registered users will have agreed to in registering for access to data, home institutions may require researchers and clinicians—who plan to use internal or external health data—to sign up to procedures and guidance documents such as a “Code of Conduct”, in order to bind them with the institutional rules and sanctions in this respect.

### Vouching

For the second route to registration for bona fide researchers, a person who has already been registered via their institution could corroborate another researcher’s status, as a reference. The vouching researcher would need to confirm that they know and have identified the researcher they are registering. To promote accountability and community control, registered users would be able to see who has vouched for whom. This is akin to having a witness to one’s competence and professional activity. The issue here is one of validation based solely on a personal statement and of potential liability for the researcher registering this way as they may not have institutional backup. “Vouchers” could also potentially be held liable. It is worth noting that a large-scale, successful community, the Debian community, maintains operating system software using a vouching approach based on Pretty Good Privacy (PGP) key signing. A member of the community must have their PGP public key signed by at least one existing member of the community before their key can be admitted into the Debian keyring (which then enables them to modify and upload software, participate in elections, etc.). There are strict guidelines on the level of proof required for signature—meeting in person, both parties show government photo ID, etc. There are also other similar prerequisites, such as accepting the social contract and advocation by another member, and violations result in removal of access by the community.

By providing several routes to registration, we hope to enable access to as wide a group of potential data users as possible while maintaining a strong level of accountability. For clinical care professionals in the USA, the National Provider Identifier issued by the Centers for Medicare and Medicaid Services could be requested in addition to the registered user’s license number. As examples, in the UK, users could provide their General Medical Council licence number; in Germany, their Lebenslange Arztnummer; in France, their numéro RPPS (répertoire partagé des professionnels de santé); in Australia, their Australian Health Practitioner Regulation Agency registration number; and in Canada, their Royal College of Physicians and Surgeons identification number. Registration for clinical care professionals will in most cases be linked to professional oversight and disciplinary governance frameworks.

In case these routes did not allow registration of atypical potential users, as an additional route to registration, any individual would also be able to apply to a standard Data Access Committee (DAC), the committees that oversee access to controlled access data, to be assessed on a case-by-case basis for registered access status. DACs may also help register users whose organizations have yet to establish the organizational or technical protocols to facilitate registration (see discussion under “Accessibility” below).

## Attestation

**Fig. 1:**
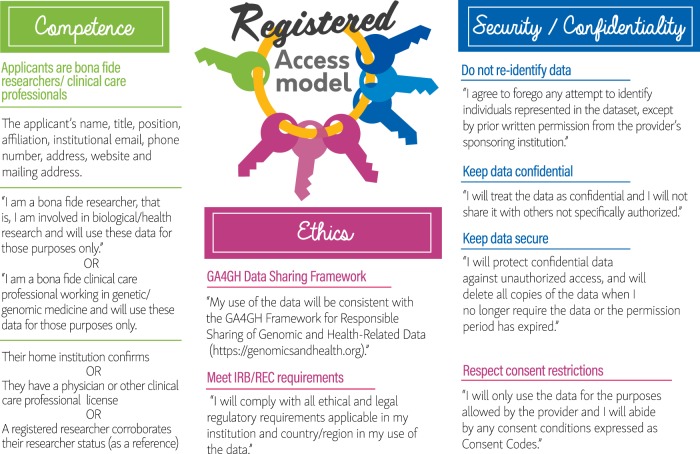
The registered access policy model. The figure shows the authentication and attestation requirements of the GA4GH registered access policy model for the user categories of bona fide researcher and clinical care professional. The seven statements shown in quotation marks form the attestation stage of the process

Integral to the definitions of bona fide researcher and clinical care professional are the statements and agreements included in the attestation stage of the registration process (see Fig. 1). Indeed, controlling the purpose of data use is a key component of data protection principles and the European Union (EU) General Data Protection Regulation (GDPR) [31].

One of the attestation statements refers to respecting consent-based data use permissions and restrictions, which should ideally be expressed as Consent Codes [32]. The GA4GH Consent Codes are a structured way of recording consent permissions so they can be made clear to users and to enable maximum data aggregation (with the same or broader permissions). Another attestation statement prohibits any attempt to identify individuals based on combining shared data with other public or non-public data sources. It allows for exceptions to this condition in some circumstances, with “prior written permission of the provider’s sponsoring institution.” This is to enable the recontact of participants if warranted (e.g., for the return of individual research results) or for permission to conduct research into privacy risks. We plan to provide general guidance for the attestation statement about keeping data secure, such as that the data should be kept encrypted at rest and in transit between systems, and that only authorized individuals have access to the keys (http://genomicsandhealth.org/work-products-demonstration-projects/security-infrastructure). We also plan to include an educational module as part of the registration process. Ultimately, we aim to enable a single format for the registration process but support a model that would allow for additional attestation statements attaching extra conditions of use for some datasets or for data from some providers. For example, Australian Genomics is considering the model that researchers need to supply proof of Human Research Ethics Committee (HREC) approval (HREC number, title, etc.), which can then be easily verified by web search.

In agreeing on these definitions of bona fide researcher/clinical care professional and level of security, cross-federation becomes possible (e.g., to enable European bona fide researchers to present queries to US Beacons at the registered access level and vice versa). The current attributes chosen to define registered users in these categories are designed to cover most of the use cases. When exceptions arise, there would have to be a very strong need for the new definition to make everyone deploy it (and populate values to the existing users retrospectively). Importantly, such exceptions would only be considered valid if driven by informed consent requirements or national laws.

Although our model does not include a review and approval of the user’s specific research or data use plans, we considered requesting abstracts of general planned data use in lay terms that would be published to enhance transparency. This may be reconsidered, especially to reinforce registered users’ commitments to using data for appropriate research or clinical care purposes and to further the aims of public transparency. Another interesting suggestion regarding transparency was to request and publish links to public researcher profiles for all registered researchers.

## Authorization

In agreeing on the proposed routes to registration, we have effectively delegated the authorization of registered users for the two categories described here to established professional employment, accreditation, or accomplishment. The data sharing environment is therefore assumed from individuals’ bona fides (including work practices and the security aspect) along with the basic set of requirements set out in the registration attestation. Along with efforts to automate registered access, this potentially limits the amount of manual authorization that will be required.

Our pilot implementation of the first registration route for academic researchers (their home institution confirms they are researchers) is the simplest in terms of liability for the category of “bona fide researchers,” and therefore the “safest” place to start. ELIXIR is piloting an approach where an ELIXIR user authenticates their identity through their own research organization’s account, and the organization confirms researcher's status. Organizational validation is assumed to improve the provenance of the researcher’s professional status because home organizations are vetted by funders, are expected to know their researchers, and can also provide the authentication credentials securely to their researchers. A challenge will be to define the requirements an organization needs to meet to become trusted in a global GA4GH registered access system (e.g., for federated identity in research, the UK has minimal checks https://www.ukfederation.org.uk/content/Documents/EligibleOrganisations).

Our experience is that this is often a more controversial and difficult challenge than the verification of individuals’ identity and role. For instance, institutions that might oversee clinicians and researchers wanting access to genetic data could include a range of clinical genetics centers (publicly funded/charitable/private); primary care centers, which treat certain inherited conditions and other contexts in which genetic testing may be commissioned or communicated without genetics specialists; and research institutions (university/other public/charitable/private). The challenge, therefore, may be to establish standards for those entities facilitating registration, including the institutions hosting registered users. A particularly crucial element of the institutional aspect of access control is the identification of accounts that no longer meet the access criteria. There needs to be well-defined, well-understood mechanisms for reviewing and revoking status, and registries of users will need to demonstrate that they successfully ensure sponsors do so in a timely manner. Examples of situations which access control workflows may need to account for include staff moving from one role to another (which may alter the user’s clinical care professional vs. researcher category) or leaving the profession.

From a technical point of view, we split the registered access architecture into two components, which can be separated organizationally and geographically: a component that manages the individual’s identity and attributes, and the party that relies upon this component to confirm identity and attributes. The OpenID Connect technical standard (http://openid.net/connect/) refers to these two components as the “OpenID Provider” and the “relying party” respectively. There may be several registries and relying parties managed by different organizations in different geographical locations.

The OpenID Provider is responsible for authenticating a registered user’s identity and for sharing attributes that the relying party may use to authorize access (see Box 2 and Fig. 1). Given OpenID Connect’s broad use worldwide, we suppose that organizations such as ELIXIR in Europe or NCBI in the US could deploy the technology needed to operate as an OpenID Provider for their constituencies.

Registered access relying parties are the entities that consume OpenID authorizations and enforce access rights and privileges based on the registered access status and attributes of the users. To be able to use registered access claims, a relying party needs to trust one or several OpenID Providers. In order to establish a federation of OpenID Providers and relying parties, they need to agree on the exact semantics of registered access status and attributes; how credentials are verified by the OpenID Provider and expressed to the relying party; what technical protocols are used to share between the registry and the relying party; and how to protect the confidentiality, integrity, and availability of the communication.

User attributes and attestations are provided to relying parties through the standardized OpenID Connect protocol, which is based on OAuth 2.0 [33]. These standards provide a mechanism through which OpenID Providers may authenticate users and provide “claims”—data structures that encode various user attributes—that can be cryptographically validated by relying parties and used in mediating access to data. Once identity has been authenticated and registered access attributes shared, OAuth 2.0 will mediate the requested access based on the data holder’s access policy.

The GA4GH is working to define a set of custom claims for registered access that all OpenID Providers and relying parties can adopt (Library Cards [34]) providing interoperability across the ecosystem of registered access adopters. Strong identity-proofing will be required within a unified identity framework, especially in the future, for registration that is independent of institutional listing or peer vouching. We plan to use existing guidelines [35] for how to establish and maintain trust in digital identities. These frameworks rank a spectrum of assurance levels, and relying parties can report (in claims) which of these levels was used to perform identity proofing.

Researcher attributes and registered access status count as personal, identifiable information, which is protected by privacy laws, including the new GDPR in the EU. To protect the privacy of researchers and respect data protection laws, it is proposed that OpenID Providers limit the amount of personal data shared with relying parties. This would mean communicating only a pseudonymous identifier of the researcher (i.e., an alphanumeric code, which is needed for thwarting re-identification and other attacks on multiple relying parties simultaneously) and their registered access status (which is needed for verifying the requestor’s status), including its route and provenance, i.e., which registry delivered the status. Consent is one of the six lawful bases to process personal information in the GDPR [36]. Article 4(11) defines consent as: “any freely given, specific, informed and unambiguous indication of the data subject’s wishes by which he or she, by a statement or by a clear affirmative action, signifies agreement to the processing of personal data relating to him or her”. For the registration process, this would entail providing users with a way to consent to the sharing of their personal data for the purposes of gaining registered status, which ELIXIR has integrated into its pilot system.

Different datasets, even within an institution, may have different requirements, such as the Consent Codes associated with data. Such datasets may require additional Attestation statements, beyond those recommended by GA4GH, for access (see Fig. 1); a data steward [37] (or the data custodian or guardian as referred to in different locations) must specify and enable such Attestations, and they will usually be guided by research ethics committees and institutional review boards in these responsibilities. While access conditions must reflect the use permissions of the dataset, additional Attestations/restrictions may complicate or prevent the aggregation of data from many sources.

## Accessibility

In the interests of efficiency and alleviating administrative burden on data custodians—particularly given the number of potential registered users—efforts should be made to automate the registered access process. Additionally, from an information security perspective, self-asserted attributes provide little accountability and raise the possibility of identity theft. We therefore sought to incorporate automated (or delegated, e.g., institutional) checks of user attributes. As our plans for the processing of registered access attributes for bona fide researcher registration draw on pre-existing academic infrastructure, we envisage minimal investment from an institutional perspective, reducing barriers to adoption of this system. It will be important to install a comparable system for access by researchers in industry.

Since 2005, research and education institutions have been operating technical frameworks called identity federations that allow researchers to use their home institution’s credentials (such as user accounts and passwords) to access services that are outside their home institutions. To register their bona fide researcher status and make the related attestations within such federations, a researcher would first need to log in at their home institution, which then delivers their fresh and validated role and affiliation information to the registration process. Currently, there is some form of national research and education identity federation in 72 countries (https://refeds.org/federations) using many different systems but usually the same technology and that are bridged with a system called eduGAIN (https://www.edugain.org; a sister service of eduroam, https://www.eduroam.org/). A benefit of using an identity federation for registered access is that the researcher status is not self-asserted by the researcher but instead claimed by the research institution employing the researcher. The home institution is also able to provide more fine-grained information on the person’s affiliation (http://software.internet2.edu/eduperson/internet2-mace-dir-eduperson-201602.html#eduPersonAffiliation) than a simple institutional e-mail address check, which often does not differentiate between researchers, students, and administrative staff. Additional details that could support registered access through federated identity management would be the categorization of bio/health researchers or even “registered following GA4GH standards.” A challenge of identity federation is that currently there is no widely deployed framework for the level of assurance of the identity and authentication of users. Data protection laws also make some institutions hesitate to release researchers’ personal data to other jurisdictions. Collaborations such as the Federated Identity Management for Research Collaboration (FIM4R) aim to establish common standards that meet the needs of various research communities [38, 39].

## Conclusion

While there remain many challenges in implementing registered access, especially at scale and with respect to the legal and administrative tools to facilitate registration through the proposed range of routes, the GA4GH pilots have allowed us to flesh out various aspects and better understand its practical utility. The main goal of registered access is to streamline access to datasets that require acceptance of terms and conditions due to consent agreements or because of a level of ethical and legal risk, and to enable access to multiple datasets at once as well as to facilitate data discovery and use. We also envisage that the simplicity, and clarity, of the standard conditions of data access and use in registered access (the attestation) will both encourage greater use of the data and respect for its ethical use, as seen with licensing terms, such as GNU General Public License and Creative Commons.

The registered access model and services described above must correctly maintain protections that were agreed to by study participants as well as researchers and clinicians who wish to study their data in order to eventually advance biomedical knowledge and benefit society. The registered access policy model will then need to be recognized and supported by many stakeholders, including research ethics boards, such that the language used in consent forms and research agreements are compatible with this access model. This will make a big difference in how “*silo*-ed” data continue to be. Ultimately, the confidence the research community will gain in the system will determine the extent of the resources it will ultimately provide.

Finally, while we have focused initially on registration criteria for researchers and clinical care professionals, many of whom have not generally had access through the controlled access system, we anticipate that data users will eventually include members of the public, including patients and citizen scientists (see e.g., mPower [40]), as well as other groups such as volunteer health-care providers and journalists. We plan to consider expanding registered access for these important and diverse groups in the near future, within the permissions of consent, and ethical standards, and with broad consultation with patient advocacy groups and research participants.

Another important aspect of improving data access is the development of ethics tools to support the assessment of data sensitivity and therefore the risk in data sharing to better determine proportionate levels of protection (e.g., open or registered). A coherent approach involves considering both the risk of re-identification of data and its sensitivity, along with the data sharing expectations of individuals and communities (Data Sharing Privacy Test [41]).

We expect registered access will inform and may even replace many controlled access mechanisms as the level of accountability that it can achieve is demonstrated over time. Data Access Committees may come to play new roles, such as deciding which data are suited to registered access, as well as reviewing applications of atypical potential users and handling other aspects of data governance (e.g., data use breaches or retractions).

We believe that it is ethically desirable to use less restrictive access controls, wherever suitable, to increase the chances of having the best research from the most people using the data that has been contributed. To needlessly reduce appropriate access likely undermines the intentions and desires of research participants as well as hindering the course of research progress.
